# Associations with sub-optimal clinic attendance and reasons for missed appointments among heterosexual women and men living with HIV in London

**DOI:** 10.1007/s10461-022-03681-x

**Published:** 2022-05-10

**Authors:** A R Howarth, V Apea, S Michie, S Morris, M Sachikonye, C H Mercer, A Evans, V C Delpech, C Sabin, F M Burns

**Affiliations:** 1 Institute for Global Health, University College London, London, UK; 2 Barts Health NHS Trust, London, UK; 3 Centre for Behaviour Change, University College London, London, UK; 4 Department of Applied Health Research, University College London, London, UK; 5 HIV i-Base, London, UK; 6 Royal Free London NHS Foundation Trust, London, UK; 7 UK Health Security Agency, London, UK; 8 UCL Institute for Global Health, Mortimer Market Centre, off Capper Street, WC1E 6JB London, UK

**Keywords:** Patient engagement, HIV, Heterosexual, Social determinants, Cross-sectional survey

## Abstract

Poor engagement in HIV care is associated with poorer health outcomes and increased mortality. Our survey examined experiential and circumstantial factors associated with clinic attendance among women (n = 250) and men (n = 106) in London with heterosexually-acquired HIV. While no associations were found for women, among men, sub-optimal attendance was associated with insecure immigration status (25.6% vs. 1.8%), unstable housing (32.6% vs. 10.2%) and reported effect of HIV on daily activities (58.7% vs. 40.0%). Among women and men on ART, it was associated with missing doses of ART (OR = 2.96, 95% CI:1.74–5.02), less belief in the necessity of ART (OR = 0.56, 95% CI:0.35–0.90) and more concern about ART (OR = 3.63, 95% CI:1.45–9.09). Not wanting to think about being HIV positive was the top reason for ever missing clinic appointments. It is important to tackle stigma and the underlying social determinants of health to improve HIV prevention, and the health and well-being of people living with HIV.

## Introduction

Health inequalities persist across the United Kingdom (UK), underpinned by variations in life conditions that contribute towards the poorer health outcomes experienced by more disadvantaged members of society [1–4]. These adverse social, cultural, political, economic, commercial and environmental conditions, or wider social determinants of health [5], are particularly pervasive among people living with HIV. A national survey of people living with HIV in England and Wales found that three quarters of Black Africans and half the people from other minority ethnicities did not always have enough money to meet their basic needs [6]. Research in the US has shown that people at socio-economic disadvantage [7] and those living in poverty [8] are disproportionately affected by HIV, and UNAIDS continues to emphasise the impact of poverty and marginalisation on HIV globally [9].

HIV is now a manageable long-term condition and successful treatment with antiretroviral therapy (ART) leads to a life expectancy that is similar to the general population [10]. ART is also an effective means of HIV prevention as it stops transmission of the virus through the suppression of HIV viraemia to undetectable levels [11]. However, the individual and public health benefits of ART can only be achieved if people with HIV are aware of their status and have sustained engagement with care and treatment. Poor engagement in HIV care is associated with a detectable viral load and poorer health outcomes [12–14], including increased mortality [15–18].

A better understanding of the factors associated with engagement in HIV care and the impact of adverse experience and circumstance on clinic attendance is therefore essential. Studies from Europe and the USA indicate that people who are male, older, White and men who have sex with men (MSM) are less likely to disengage from HIV care [19–24]. Studies also indicate that those with more complex needs, such as intravenous drug users and migrants, are more likely to disengage from care [20, 22, 24]. Stigma, isolation, poverty and adverse social circumstances are significant barriers to engaging in care and living well with HIV [25–29] and those at socio-economic disadvantage are less likely to prioritise their HIV care [30].

The REACH project (Retention and Engagement Across specialised Care services for HIV) set out to understand patterns of HIV outpatient attendance among people with HIV to develop cost effective interventions to optimise engagement in care [31]. We conducted a cross-sectional survey of people attending HIV outpatient clinics in London, UK. Our survey was developed to cover all components of the COM-B model, a theoretical framework which proposes that behaviour is the outcome of an interaction between an individual’s capability, opportunity and motivation [31, 32].

We found that those who had greater difficulties engaging with HIV services were younger, had been diagnosed for longer, were less likely to be home-owners or registered with a general practitioner (primary care physician), and were more likely to have children, to report neurocognitive impairment and poorer health, and have clinic-reported drug or alcohol dependency [31].

Given the reported differences in engagement between women and men and according to route of HIV transmission, in this paper, we concentrate on the sub-sample of women and men from REACH with heterosexually-acquired HIV. The intervening period since our survey was fielded in 2014–2015 has seen important changes in our understanding of the virus that have impacted HIV care, including new evidence that adherence to ART means that HIV cannot be sexually transmitted to others. We believe, however, that our use of these data to examine the social determinants of health for people living with HIV remains highly relevant. Here, we focus on experiential and circumstantial factors to explore the potential impact of the wider social determinants of health on HIV clinic attendance, explore the association between clinic attendance and current HIV treatment and health status, and examine the reasons participants give for missing their appointments at the HIV clinic.

## Methods

### Study design

A cross-sectional survey of people attending HIV outpatient clinics in London, UK.

### Setting and sampling

Participants were recruited from seven HIV clinics in London (May 2014 – August 2015) and classified according to their clinic attendance. People with optimal attendance had attended all their HIV clinic appointments in the past year. UK guidelines at the time of the study indicated that patients should be seen within 2–4 weeks of starting ART and every 3–6 months for routine monitoring if on ART and considered medically ‘stable’ [33]. We applied a simple algorithm that could be used by research staff in the seven clinics to recruit patients according to their recent attendance behaviour. Regardless of the complexity of their case / regularity of their appointments, people were classified as having sub-optimal attendance if they had missed at least one HIV clinic appointment in the past year (which had not been re-booked within four weeks), or had experienced a period of non-attendance for a year or more that had ended within the past year. This is based on evidence that one missed visit in the first year of HIV care is associated with increased risk of mortality [16].

People identifying as “female” or “male” who acquired HIV through heterosexual transmission (as recorded in their clinical notes) were selected from the overall sample to be included in the following analysis.

### Data collection

Local research staff systematically approached clinic attendees in order to achieve a sample of at least 100 people with optimal attendance in the past year and 100 with sub-optimal attendance. Written informed consent was obtained and no financial incentive was offered for participation. The anonymous self-completion pen-and-paper questionnaire contained 80 questions and took 20–30 min to complete. Questionnaire responses were linked to clinical data.

### Measures

Questions on date of birth, ethnic group, country of birth, immigration status, relationship status, number of children, housing, employment, education and HIV diagnosis were included. Participants were asked to report the importance of religion in their lives, whether HIV affected their day-to-day activities, who they had told about their HIV, whether they had enough money for their basic needs and their experience of intimate partner violence. Items on recreational drug use were included. They were asked whether they missed appointments because of drinking alcohol, taking drugs or to look after children or others, and to tick all applicable items from a list of reasons for missed appointments. Each reason was rated on a 4-point Likert scale from “Never” to “Often”. Those on ART were asked how many doses they had missed in the past week. Women were asked if they were currently pregnant, had given birth in the past year or had been diagnosed during pregnancy.

Items from the following scales were included in the questionnaire: the Patient Health Questionnaire (PHQ) [34], with reported symptoms rated as normal (0–2), mild (3–5), moderate (6–8), and severe (9–12); the Strive Internalised Stigma scale [35], with stigma rated as low for 1–3 items ticked and high for 4–7 items ticked; the Duke-UNC Social Support Questionnaire [36] with low social support rated for a score of ≤ 12; and the Beliefs about Medicines Questionnaire (BMQ) [37, 38], including the *ART necessity* and *ART concerns* sub-scales which ranged from 4 (low) to 20 (high).

Clinics collected data on ART, CD4 count, viral load, drug / alcohol dependency and patient complexity according to the HARS 3 category [39].

### Data analysis

Only women and men who acquired HIV heterosexually were included in the analysis. The chi squared test was used to examine differences between women and men, with Fisher’s Exact test used when expected values in any cells were small.

Binary logistic regression was used to analyse associations between predictor variables and clinic attendance and to test for an interaction with gender. Variables were selected for inclusion in multivariable logistic regression models if they were significantly associated with attendance in univariate analysis (p < .05).

#### Ethical approval

Ethical approval for the study was obtained from the National Research Ethics Service Committee London – City Road & Hampstead (reference 14/LO/0039).

A more detailed description of the study methodology is provided elsewhere [31].

## Results

The overall sample included 983 individuals, with 36.2% (356/983) having acquired HIV heterosexually. Among these 356 participants, 191 (53.7%) had attended all their appointments in the past year and the attendance of the remaining 165 (46.3%) had been sub-optimal. Among those with sub-optimal attendance, 59.4% had missed ≥ 1 clinic appointment in the past year and 40.6% had a history of > 1 year of non-attendance – there was no difference by gender (χ^2^ = 0.01, d.f.=1, p = .91).

### Gender comparison

Women made up 70.2% (250/356) of the sample. There was no significant difference in attendance pattern by gender: 47.6% of women (n = 119) and 43.4% of men (n = 46) had attended sub-optimally in the past year (χ^2^ = 0.53, d.f.=1, p = .47). The women were younger than the men, with 54.0% aged 31–45 years (vs. 31.1% of men) and 40.0% aged > 45 years (vs. 64.2% of men, χ^2^ = 17.67, d.f.=2, p < .001) (Table 1). Women were more likely to be of Black African ethnicity (71.6% vs. 58.5%) and less likely to be of White (12.8% vs. 21.7%) or ‘Other’ ethnicity (15.6% vs. 19.8%, χ^2^ = 6.45, d.f.=2, p = .04). There were no significant differences by gender for region of birth or years in education. Men were more likely than women to be diagnosed with HIV at an older age, with 61.3% diagnosed aged 31–45 years (vs. 40.0% of women) and 25.5% diagnosed at ≤ 30 years old (vs. 52.0% of women, χ^2^ = 21.29, d.f.=2, p < .001) and were more likely to have a CD4 count < 200 cells/mm^3^ at diagnosis (63.4% vs. 35.4%, χ^2^ = 15.68, d.f.=2, p < .001).

**Table 1:** Socio-economic and HIV background, by gender

Characteristic	Women - n (%) [n = 250]		Men - n (%) [n = 106]	χ^2^ value	p value
**Socio-economic background**					
Current age group					
*30 years and under*	15 (6.0)		5 (4.7)	17.67	< 0.001
*31 to 45 years*	135 (54.0)		33 (31.1)		
*Over 45 years*	100 (40.0)		68 (64.2)		
Ethnic group					
*Black African*	174 (71.6)		62 (58.5)	6.45	0.04
*White*	31 (12.8)		23 (21.7)		
*Other ethnic group*	38 (15.6)		21 (19.8)		
Region of birth					
*Africa*	182 (72.8)		67 (64.4)	2.34	0.27
*UK*	34 (13.6)		17 (16.3)		
*Other*	34 (13.6)		20 (19.2)		
Education after 16 years					
*None*	41 (17.7)		18 (19.8)	3.01	0.22
*Up to 2 years*	31 (13.4)		6 (6.6)		
*3 years or more*	159 (68.8)		67 (73.6)		
**HIV background**					
Age group at diagnosis					
*30 years and under*	130 (52.0)		27 (25.5)	21.29	< 0.001
*31 to 45 years*	100 (40.0)		65 (61.3)		
*Over 45 years*	20 (8.0)		14 (13.2)		
CD4 count at diagnosis					
*<200 cells/mm* ^*3*^	57 (35.4)		45 (63.4)	15.68	< 0.001
*200–349 cells/mm* ^*3*^	54 (33.5)		14 (19.7)		
*≥350 cells/mm* ^*3*^	50 (31.1)		12 (16.9)		
10 + years since HIV diagnosis	139 (55.6)		64 (60.4)	0.69	0.41

We examined differences between women and men on all independent variables included in the subsequent analysis (the variables included in Tables 2 and 4). The only significant differences (p ≤ .05) were that women were more likely to report that religion was very important to them (71.3% vs. 56.8%, χ^2^ = 5.04, d.f.=1, p = .03) and to have children (74.1% vs. 61.3%, χ^2^ = 5.79, d.f.=1, p = .02), and men were more likely to report that they had used recreational drugs in the past year (14.9% vs. 3.4%, χ^2^ = 14.71, d.f.=1, p = .001).

Only 2.0% of the women were pregnant at time of survey completion and 2.9% had given birth in the previous year. More than one quarter (27.0%) of the women had been diagnosed with HIV during pregnancy.

**Table 2:** Socio-economic and HIV background, current experience and circumstances, by clinic attendance

	All		Optimal*		Sub-optimal*					
**Characteristic**	**n**	**%**	**n**	**%**	**n**	**%**	**OR**	**(95% CI)**	**p value**	**p value [Wald] for interaction with gender**
**Socio-economic background**										
Gender										
*Female*	250	70.2	131	68.6	119	72.1	1		0.47	
*Male*	106	29.8	60	31.4	46	27.9	1.19	(0.75–1.81)		
Current age group										
*30 years and under*	20	5.6	8	4.2	12	7.3	1		0.002	0.65 [0.87]
*31 to 45 years*	168	47.2	76	39.8	92	55.8	0.81	(0.31–2.08)	0.66	
*Over 45 years*	168	47.2	107	56.0	61	37.0	0.38	(0.15–0.98)	0.05	
Ethnic group										
*Black African*	236	67.6	129	69.0	107	66.0	1		0.75	0.83 [0.37]
*White*	54	15.5	29	15.5	25	15.4	1.04	(0.57–1.88)	0.90	
*Other ethnic group*	59	16.9	29	15.5	30	18.5	1.25	(0.71–2.21)	0.45	
Region of birth										
*Africa*	249	70.3	140	73.3	109	66.9	1		0.40	0.12 [4.26]
*UK*	51	14.4	24	12.6	27	16.6	1.45	(0.79–2.64)	0.23	
*Other*	54	15.3	27	14.1	27	16.6	1.28	(0.71–2.32)	0.41	
Education after 16 years										
*None*	59	18.3	31	17.9	28	18.8	1		0.98	0.71 [0.68]
*Up to 2 years*	37	11.5	20	11.6	17	11.4	0.94	(0.41–2.15)	0.89	
*3 years or more*	226	70.2	122	70.5	104	69.8	0.94	(0.53–1.68)	0.84	
**HIV background**										
Age group at diagnosis										
*30 years and under*	157	44.1	68	35.6	89	53.9	1		0.001	0.19 [3.33]
*31 to 45 years*	165	46.3	98	51.3	67	40.6	0.52	(0.34–0.81)	0.004	
*Over 45 years*	34	9.6	25	13.1	9	5.5	0.28	(0.12–0.63)	0.002	
CD4 count at diagnosis										
*<200 cells/mm* ^*3*^	102	44.0	60	48.0	42	39.3	1		0.24	0.80 [0.45]
*200–349 cells/mm* ^*3*^	68	29.3	37	29.6	31	29.0	1.20	(0.64–2.22)	0.57	
*≥350 cells/mm* ^*3*^	62	26.7	28	22.4	34	31.8	1.74	(0.92–3.28)	0.09	
10 + years since HIV diagnosis	203	57.0	110	57.6	93	56.4	0.82	(0.62–1.45)	0.82	0.24 [1.41]
**Current experiences and circumstances**										
Immigration status										
*British citizen*	186	52.7	97	56.1	89	58.6	1		0.41	0.02 [10.02]
*EU citizen*	37	11.4	22	12.7	15	9.9	0.74	(0.36–1.52)	0.42	
*Permanent residency*	63	19.4	37	21.4	26	17.1	0.77	(0.43–1.37)	0.37	
*Non-permanent*	39	12.0	17	9.8	22	14.5	1.41	(0.70–2.83)	0.33	
Homeless or temp housing	65	18.6	33	17.6	32	19.8	1.16	(0.67–1.98)	0.60	0.003 [8.88]
Insufficient money	70	20.1	35	18.4	35	22.0	1.25	(0.74–2.11)	0.41	0.12 [2.45]
Working FT or PT ^§^	160	47.3	85	46.7	75	48.1	1.06	(0.69–1.62)	0.80	0.92 [0.01]
Relationship status										
*Not in relationship*	170	50.6	98	52.7	72	48.0	1		0.57	0.27 [2.60]
*Yes – not co-habiting*	64	19.0	32	17.2	32	21.3	1.12	(0.68–1.83)	0.66	
*Yes – co-habiting*	102	30.4	56	30.1	46	30.7	1.36	(0.77–2.42)	0.30	
IPV: Emotional abuse ^‡^	50	18.9	28	19.2	22	18.6	0.97	(0.52–1.80)	0.91	0.82 [0.05]
IPV: Afraid of partner ^‡^	40	15.2	26	17.8	14	11.9	0.62	(0.31–1.25)	0.18	0.34 [0.93]
Low social support	59	19.5	32	19.8	27	19.3	0.97	(0.55–1.72)	0.92	0.93 [0.01]
Strong religion	169	67.1	92	68.1	77	65.8	0.90	(0.53–1.52)	0.69	0.64 [0.22]
Has children	248	70.3	128	67.4	120	73.6	1.35	(0.85–2.15)	0.20	0.63 [0.23]
Told no one about HIV	70	20.2	34	18.3	36	22.5	1.30	(0.77–2.20)	0.33	0.86 [0.03]
Internalised stigma										
*None reported*	132	38.5	77	41.4	55	35.0	1		0.26	0.37 [1.97]
*Low (1–3 items)*	170	49.6	91	48.9	79	50.3	1.21	(0.77–1.92)	0.41	
*High (4–7 items)*	41	12.0	18	9.7	23	14.6	1.79	(0.88–3.63)	0.11	
HIV affects day-to-day activity										
*No*	183	52.0	97	51.3	86	52.8	1		0.54	0.03 [7.10]
*Yes, a little*	100	28.4	51	27.0	49	30.1	1.08	(0.67–1.77)	0.75	
*Yes, a lot*	69	19.6	41	21.7	28	17.2	0.77	(0.44–1.35)	0.36	
PHQ4: anxiety and depression (past 2 weeks)										
*Normal*	231	74.0	128	75.7	103	72.0	1		0.19	0.29 [2.45]
*Mild*	41	13.1	17	10.1	24	16.8	1.75	(0.90–3.44)	0.10	
*Moderate*	40	12.8	24	14.2	16	11.2	0.83	(0.42–1.64)	0.59	
Recreational drug use (past year)	23	6.8	10	5.3	13	8.6	1.67	(0.71–3.92)	0.24	0.11 [2.54]
Drug / alcohol dependency (past year)	13	3.9	4	2.2	9	5.9	2.78	(0.84–9.22)	0.09	0.67 [0.19]

* In the past year

^§^ FT: full-time; PT: part-time

^‡^ IPV: Intimate partner violence

### Associations with sub-optimal attendance

We examined the association between variables relating to socio-economic and HIV background, current experience and circumstances and clinic attendance (Table 2). In unadjusted analyses, the only significant differences between participants with sub-optimal and optimal attendance, were by current age and age at diagnosis, with some indication of an effect of CD4 count at diagnosis and drug / alcohol dependency. Compared to those aged < 30 years at the time of the survey, those aged > 45 years were less likely to have sub-optimal attendance (OR = 0.38, 95% CI: 0.15–0.98, p = .05) but there was no significant difference for those aged 31–45 years (OR = 0.81, 95% CI: 0.31–2.08, p = .66). Compared to participants aged < 30 years at HIV diagnosis, those diagnosed at older ages were less likely to have sub-optimal attendance (31 to 45 years, OR = 0.52, CI: 0.34–0.81, p = .004 and > 45 years, OR = 0.28, 95% CI: 0.12–0.63, p = .002). Compared to those with a CD4 count < 200 cells/mm^3^ at diagnosis, participants with a CD4 count ≥ 350 cells/mm^3^ at diagnosis were more likely to have sub-optimal attendance (OR = 1.74, 95% CI: 0.92–3.28, p = .09) and those with clinic-reported drug and / or alcohol dependency in the past year were also more likely to have sub-optimal attendance (OR = 2.78, 95% CI: 0.84–9.22, p = .09).

We examined the same background, current experience and circumstances variables to test for interactions with gender (Table 2). We found significant interactions for immigration status (Wald = 10.02, p = .02), housing status (Wald = 8.88, p = .003) and impact of HIV on daily activity (Wald = 7.10, p = .03). Associations between these variables and attendance are presented separately for women and men in Table 3. Among women, there were no significant differences in attendance pattern by immigration, housing or daily activity. However, among men, those with sub-optimal attendance were significantly less likely to have British or EU citizenship or permanent residency in the UK, with lack of secure, long-term immigration status reported by 25.6% of those with sub-optimal attendance compared to 1.8% of those with optimal attendance (χ^2^ = 13.95, d.f.=3, p = .003). Those with sub-optimal attendance were also more likely to report insecure housing (32.6% vs. 10.2%, χ^2^ = 8.13, d.f.=1, p = .004) and to report no effect of HIV on their day-to-day activities (60.0% vs. 41.3%, χ^2^ = 5.93, d.f.=2, p = .05).

**Table 3:** Association between clinic attendance and immigration status, insecure housing and daily activity, by gender

	Women						Men				
**Characteristic**	**All** **n (%)** **[n = 250]**	Optimal* **n (%)** **[n = 131]**	Sub-optimal* **n (%)** **[n = 119]**	**χ**^**2**^ **value**	**p value**		**All** **n (%)** **[n = 106]**	Optimal* **n (%)** **[n = 60]**	Sub-optimal* **n (%)** **[n = 46]**	**χ**^**2**^ **value**	**p value**
Immigration status											
*British citizen*	135 (59.0)	63 (54.3)	72 (63.7)	2.52	0.47		51 (53.1)	34 (59.6)	17 (43.6)	13.95	0.003
*EU citizen*	21 (9.2)	13 (11.2)	8 (7.1)				16 (16.7)	9 (15.8)	7 (17.9)		
*Permanent residency*	45 (19.7)	24 (20.7)	21 (18.6)				18 (18.8)	13 (22.8)	5 (12.8)		
*Non-permanent*	28 (12.2)	16 (13.8)	12 (10.6)				11 (11.5)	1 (1.8)	10 (25.6)		
Homeless or temp housing	44 (18.0)	27 (20.9)	17 (14.7)	1.63	0.20		21 (20.0)	6 (10.2)	15 (32.6)	8.13	0.004
HIV affects day-to-day activity											
*No*	128 (52.0)	61 (47.3)	67 (57.3)	2.57	0.28		55 (51.9)	36 (60.0)	19 (41.3)	5.93	0.05
*Yes, a little*	71 (28.9)	40 (31.0)	31 (26.5)				29 (27.4)	11 (18.3)	18 (39.1)		
*Yes, a lot*	47 (19.1)	28 (21.7)	19 (16.2)				22 (20.8)	13 (21.7)	9 (19.6)		

* In the past year

We conducted multivariable binary logistic regression, including variables significantly associated (p ≤ .05) with attendance among men in the above analysis (age at diagnosis, immigration status, housing status and impact of HIV on daily activity). We did not conduct a multivariable analysis for women, as we found no significant associations in the above analysis. Among men, attendance was independently associated with age at diagnosis and immigration status. Compared to men in the 30 years and under age group, the older age groups were less likely to have sub-optimal attendance (31–45 years: aOR = 0.25, 95% CI: 0.08–0.80, p = .02; >45 years: aOR = 0.17, 95% CI: 0.03–1.05, p = .06). Compared to those with British citizenship, men with insecure immigration status were more likely to have sub-optimal attendance (aOR 21.6 95% CI 2.25–207.8, p = .008).

We examined current health status, treatment behaviour and beliefs for women and men (Table 4). We found that those with sub-optimal attendance were more likely than those with optimal attendance to have a detectable viral load (OR = 4.32, 95% CI: 2.59–7.22, p < .001) and complex healthcare needs (OR = 1.95, 95% CI: 1.18–3.21, p = .009). They were less likely to be on ART (OR = 0.25, 95% CI: 0.11–0.56, p = .001). Among those on ART, people with sub-optimal attendance were more likely to have missed a treatment dose of ART in the past week (OR = 2.96, 95% CI: 1.74–5.02, p < .001). They were less likely to report strong belief in the necessity of ART (OR = 0.56, 95% CI: 0.35–0.90, p = .02) and were more likely to report concerns about ART (low vs. medium concern: OR = 1.54, 95% CI: 0.93–2.54, p = .09; low vs. high concern: OR = 3.63, 95% CI: 1.45–9.09, p = .001). There were no significant interactions with gender on any of these variables.

**Table 4:** Current health status, treatment behaviour and beliefs, by clinic attendance

	All		Optimal*		Sub-optimal*					
**Characteristic**	**n**	**%**	**n**	**%**	**n**	**%**	**OR**	**(95% CI)**	**p value**	**p value [Wald] for interaction with gender**
Detectable viral load	95	26.9	27	14.2	68	41.7	4.32	(2.59–7.22)	< 0.001	0.95 [0.004]
Complex health needs ^§^	83	23.3	34	17.8	49	29.7	1.95	(1.18–3.21)	0.009	0.99 [0.00]
Currently on ART	323	90.7	183	95.8	140	84.8	0.25	(0.11–0.56)	0.001	0.42 [0.66]
Missed dose of ART (past week) ^¶^	82	28.1	31	18.7	51	40.5	2.96	(1.74–5.02)	< 0.001	0.55 [0.37]
Strong belief in necessity of ART ^¶^	154	53.7	98	59.8	56	45.5	0.56	(0.35–0.90)	0.02	0.48 [0.51]
Concerns about ART ^¶^										0.91 [0.20]
*Low*	135	48.0	87	54.0	48	40.0	1			
*Medium*	122	43.4	66	41.0	56	46.7	1.54	(0.93–2.54)	0.09	
*High*	24	8.5	8	5.0	16	13.3	3.63	(1.45–9.09)	0.001	

* In the past year

^§^ According to HARS 3

^¶^ Among participants on ART

### Reasons for missed appointments

Not wanting to think about being HIV positive was the top reason for ever missing appointments, given by both those with sub-optimal (41.3%) and optimal attendance (23.1%) in the past year (Table 5). Simply forgetting was a key reason given by those with sub-optimal attendance (41.2%), and feeling too tired and depressed or not having enough money were among the most common reasons that both groups gave for sometimes or often missing their appointments. While 30.8% of those with sub-optimal attendance ever missed appointments because they had enough medication, and 28.9% did so because they could not get time off work, these reasons were lower down on the list for those with optimal attendance in the past year (12.4% and 10.7%, respectively). There were no significant differences between women and men on the reasons for missed appointments that are listed in Table 5.

**Table 5:** Reasons given for ever missing HIV clinic appointments, ordered by frequency for sub-optimal attendance

Reason for sometimes or often missing appointment	N	Sub-optimal* n (%)	Optimal* n (%)	Rank position for optimal attendance*
Didn’t want to think about HIV	282	52 (41.3)	36 (23.1)	1
Simply forgot	316	56 (41.2)	25 (13.9)	8
Felt too tired	290	43 (33.9)	34 (20.9)	3
Felt depressed / overwhelmed	293	43 (33.6)	35 (21.2)	2
Didn’t have enough money	282	41 (32.5)	32 (20.5)	4
Had enough medication	273	37 (30.8)	19 (12.4)	10
Couldn’t get time off work	271	35 (28.9)	16 (10.7)	11
Felt too sick or ill	289	34 (27.0)	26 (16.0)	6
Felt well	267	30 (26.3)	29 (19.0)	5
Didn’t have transport	281	30 (24.4)	22 (13.9)	8
Afraid of being seen at clinic	280	26 (20.8)	22 (14.2)	7
Not followed doctor’s advice	276	24 (20.0)	7 (4.5)	12
Didn’t think doctor could help	273	17 (14.8)	6 (3.8)	13

* In the past year

Among those with sub-optimal attendance, 7.1% had missed appointments because of drinking alcohol and 5.8% because of taking recreational drugs. Among those with optimal attendance, this was reported by 2.3% and 1.3%, respectively. Men were more likely to report recreational drug use and among men with sub-optimal attendance, 16.3% had missed appointments because of taking drugs and 20.9% had done so because of drinking alcohol.

Half of the participants (52.2%) reported caring responsibilities and women were more likely to report such responsibilities (59.7% vs. 34.6%, χ^2^ = 18.32, d.f.=1, p < .001). Among men and women with caring responsibilities, 33.9% sometimes or often missed clinic appointments for this reason: 51.1% of those with sub-optimal attendance and 17.4% of those with optimal attendance in the past year. There was no significant difference between women and men who reported caring responsibilities on missing appointments for this reason (35.4% vs. 27.8%, χ^2^ = 0.75, d.f.=1, p = .39).

## Discussion

Our findings support previous work which shows that disengagement from HIV care among heterosexual women and men is more likely among those who are younger [19, 20, 23, 24]. Those with sub-optimal attendance in the past year were more likely to have a detectable viral load and complex healthcare needs, and less likely to be on ART. Among those on ART, sub-optimal attendance was associated with missing doses of ART, less belief in the necessity of ART and more concerns about taking ART. The top reason that participants gave for ever missing their appointments at the HIV clinic was related to stigma and not wanting to think about being HIV positive, which was reported by two fifths of those with sub-optimal attendance in the past year and one fifth of those who had attended all appointments.

Precarious immigration and housing were associated with sub-optimal attendance among men, and immigration status remained significantly associated with clinic attendance in multivariate analysis for men (along with age). It is also notable that about one fifth of our participants were living in insecure housing and / or did not have enough money for their basic needs. These findings highlight the importance of incorporating social prescribing (which involves referral to local, non-clinical services within the community) into holistic support for people with HIV with the aim of addressing such inequities.

We examined the influence of interpersonal factors on engagement in care. Our previous qualitative research found that partners can have a protective or destructive effect on engagement in care [31] but we did not find an association here with variables relating to partnerships, faith or social support. HIV, however, remains a highly stigmatised condition. One fifth of our participants had not told anyone (apart from healthcare professionals) about their HIV status, and about two thirds reported markers of internalised HIV stigma. Not wanting to think about being HIV positive was the most common reason given for missed appointments among both those with sub-optimal and optimal attendance. One fifth of those with sub-optimal attendance in the past year had missed appointments because they did not want to be seen at the clinic. It remains essential to tackle the causes of stigma and marginalisation among people with HIV.

One fifth of our participants had not attended appointments because they felt too depressed or overwhelmed. While HIV per se may not be the cause of depression, high levels of poor mental health have been found among people with HIV in the UK: 27% of older and 21% of younger people with HIV reported depressive symptoms compared to just 8% of the older control group [40] and people with HIV are also twice as likely as the general population to report symptoms of depression and anxiety [41]. High suicide rates are furthermore reported during the first year after diagnosis, particularly among men [42]. Other studies have found that people with HIV in the UK experience poorer health-related quality of life than the general population [43, 44] and our study supports the call to add a “fourth 90” to UNAIDS’s 90–90–90 targets for global HIV control [45] − 90% diagnosis of HIV, 90% treatment, 90% viral suppression AND 90% mental wellness [46].

Previous research has found that people who inject drugs are more likely to disengage from care [22]. Our sample excluded people who had acquired HIV from injecting drug use but clinic-reported drug / alcohol dependency was associated with sub-optimal attendance among our participants. Men were more likely to have used recreational drugs and to report missing appointments at the clinic because of recreational drug use. This reinforces the necessity of implementing measures to actively solicit drug use history, systematically identify individuals at risk, and provide mental health and addiction services in clinic [47].

Caring responsibilities were a key reason for missed clinic appointments. Women were much more likely to report such responsibilities than men (including any unpaid caring) and one third of participants with these responsibilities sometimes or often missed clinic appointments because of this. Among those with sub-optimal attendance in the past year who had such responsibilities, half reported missing their appointments for this reason. Our findings emphasise the need to address barriers to attendance, such as childcare, not having the money or time off work, which have been discussed elsewhere [48, 49]. The lack of association between background variables and attendance among women, raises concerns that routinely collected clinic data do not help to signal potential difficulties for women and brings into question whether routinely collected data should be different or more inclusive.

As previously found [12–14], those with sub-optimal attendance were more likely to have a detectable viral load and complex healthcare needs. The survey was implemented before the era of universal testing and treatment and we found an association between not being on ART and sub-optimal attendance. After treatment became a nationally commissioned service in the UK, the proportion of newly diagnosed people on treatment within three months of diagnosis rose from 53% to 2014 to 78% in 2018 [50]. We may also be optimistic about the potential for universal testing and treatment to improve engagement in care. Among those on ART, people with sub-optimal attendance in the past year were more likely to have missed a dose in the past week. They were more likely to be sceptical about the value of ART and express greater concerns about ART. Further work on managing these attitudes towards ART is needed to realise the full benefit of universal treatment.

Although we sought to recruit a representative sample of people living with HIV in London by including HIV clinics from across London (central, north, south, east and west), this study is based on a convenience sample of people attending these clinics at the time of survey implementation and does not include those who were out of care at that time. As the data were wholly collected in London, it is possible that the factors associated with clinic attendance and reasons given for missed appointments could be different outside London. However, our findings from analysing the complete dataset (including those who acquired HIV non-heterosexually) were similar to those from analysis of UK CHIC data, and are also congruent with data from the ASTRA study, which included study sites across the UK [51].

This cross-sectional survey cannot provide evidence of a causal link between the factors identified and HIV clinic attendance or assess the impact of unmeasured confounders on the analysis. It is based on a convenience sample of people attending seven HIV clinics in London and their responses to a survey designed by the research team. However, the survey was developed from the COM-B model [31, 32] and, wherever possible, incorporated validated items.

Our questionnaire was extensive, and while we have selected the relevant items here to examine associations with the social determinants of health, undertaking multiple hypothesis tests may run the risk of Type I error. While our multivariable analysis was subject to potential multicollinearity, it indicated that further exploration of *how* immigration and housing status, and day-to-day activity contribute to HIV clinic attendance may be useful.

The power calculation for REACH was based on a sample of one thousand participants and our sub-sample analysis here may not be sufficiently powered to detect significant differences between groups. In view of the reduced sample size, we combined participants who missed one or more appointments with those who did not attend for a year or more into one group and so were unable to explore any “dose effect” of missed appointments. The findings may also reflect the changed perspectives of people who have returned to care.

Our findings demonstrate the extent of the adverse experiential and circumstantial factors which affect people living with HIV and the association of these factors with engagement in HIV care. They highlight the impact of stigma on HIV clinic attendance and the need to manage patients’ attitudes towards ART in order to realise the individual and public health benefits of universal testing and treatment in the UK. They underline the importance of tackling these issues in order to improve engagement in HIV care and enhance the health and well-being of people living with HIV.

## Data Availability

Researchers who are interested in accessing the REACH survey data should contact the corresponding author with a description of their proposal and material.

## Code Availability

N/A.
